# A Frequency-Adjustable Tuning Fork Electromagnetic Energy Harvester

**DOI:** 10.3390/ma15062108

**Published:** 2022-03-12

**Authors:** Qinghe Wu, Shiqiao Gao, Lei Jin, Shengkai Guo, Zuozong Yin, He Fu

**Affiliations:** State Key Laboratory of Explosion Science and Technology, Beijing Institute of Technology, Beijing 100081, China; wuqh_123@163.com (Q.W.); guoshengkai9@126.com (S.G.); yinzuozong@163.com (Z.Y.); fuhekkxx@outlook.com (H.F.)

**Keywords:** electromagnetic, tuning fork, vibration mode, energy harvester, frequency

## Abstract

In this paper, a frequency-adjustable tuning fork electromagnetic energy harvester is introduced. The electromagnetic vibration energy harvester can adjust its natural frequency according to a change in the environmental excitation frequency without any change to the structure. In the frequency-adjustable range, it can make the energy harvester resonant with the environment excitation, and the output frequency stays the same. The frequency-adjustable tuning fork electromagnetic energy harvester significantly increases the range of frequencies used. The operating frequency of the centre can be easily switched from 9.2 to 20 Hz, enabling the application of multiple excitation frequencies. In addition, the output power and power density are significantly increased compared to a piezoelectric tuning fork energy harvester of the same size. The peak power is 23.59 mW at 9.2 Hz, the power increases by 14.85 mW, and the power density increases by 169.88%. The experimental results show that the electromagnetic tuning fork frequency-adjustable conversion energy harvester can make the LED lamp work.

## 1. Introduction

In recent years, the use of environmental vibration power generation has become favoured, and it is a very promising power supply method [1,2,3,4,5]. Typical energy harvesters studied to obtain energy from the environment are piezoelectric energy harvesters [6,7,8,9], electromagnetic energy harvesters [10,11,12] and electrostatic energy harvesters [13,14,15,16]. In addition, hybrid energy harvesters [17,18,19,20] have also been studied. Among them, electromagnetic energy harvesters are used because of their large current, small internal resistance, ease of fabrication and other characteristics [10,11].

Because environmental vibration is usually low frequency (0–30 Hz), and the natural frequency of the energy harvester is usually high, making the frequency of the energy collector resonant with the vibration of the environment is one of the most prominent problems in energy-acquisition power generation [21,22,23]. Thus, people have put forward the capture energy structure with up-frequency conversion, including the contact type and noncontact type [24,25,26,27,28,29].

The contact method is realized by mechanical means. Halim et al. [25] proposed a kind of up-conversion energy harvester. A free-moving ball periodically hits two magnets on a spring, causing them to vibrate at high frequencies. The device has non-resonant characteristics. Zhang et al. [27] proposed an up-conversion energy collector, which uses a rolling bead. The collision with the piezoelectric cantilever causes it to vibrate continuously and generate electricity.

The noncontact method of energy harvesting can be achieved by a magnetic field. Fu et al. [24] proposed an energy harvester which consists of piezoelectric beams and rotating magnets. Through coupling, energy is transferred to the piezoelectric beam. Wang et al. [29] designed a variable frequency structure that uses weights to help the pendulum adjust its natural frequency and then generates electricity through electromagnetic induction.

However, most of them contain independent drive structures and power-generation structures, which are relatively complex. Wu et al. [30] proposed a tuning-fork-type piezoelectric energy harvester, which realized the function of the self-frequency conversion of a single structure and simplified the structure. However, its frequency is not adjustable, and the applicable frequency range is narrow. The piezoelectric material internal resistance is high, and the output power is limited.

In this paper, a frequency conversion electromagnetic energy harvester with an adjustable tuning fork is proposed. A clever point is that a very simple and effective adjustment knob is designed on the tuning fork handle. Without any redesign of the structure, the frequency of the energy harvester can be easily and quickly adjusted to the same frequency as the environmental vibration.

The tuning fork structure has an in-phase vibration mode and anti-phase vibration mode. The in-phase vibration mode is the low-order vibration mode closer to the low-frequency vibration of the environment, and the anti-phase vibration mode is the high-order vibration mode, which is more suitable for power generation output. The frequency adjustment knob is designed to adjust the in-phase vibration frequency, so adjusting the frequency of the energy harvester to match the environmental vibration frequency does not affect the energy conversion and output frequency. Even if the frequency of the energy harvester is adjusted, the frequency of the output electric energy remains unchanged. Adjusting the frequency makes the energy harvester tolerant of different environmental vibration frequencies, and the power generation frequency of the energy harvester can still generate power at a fixed high frequency, which also ensures the stability of the output frequency of the energy harvester and reduces the influence on electrical equipment. 

In addition, due to electromagnetic and piezoelectric power generation, the relative internal resistance is much smaller, reducing the impact on the output power. Thus, the tuning fork frequency-conversion electromagnetic energy harvester has the advantage of low internal resistance and high power.

Based on the theory and experiment, the influence of load resistance on structural vibration is analysed and tested. The results are consistent with each other, and the influence law of load resistance on structural vibration is clarified.

## 2. Design and Working Principle

### 2.1. The Structure Design

**Figure 1:**
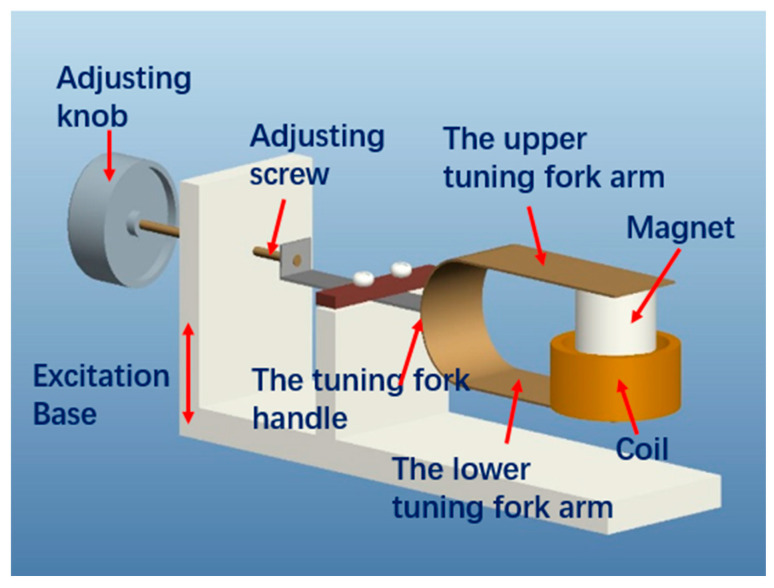
Structure of the adjustable-frequency tuning fork electromagnetic energy harvester.

The tuning fork electromagnetic energy harvester is mainly composed of a tuning fork structure, adjust screw, coil, magnet and fixed support. The magnets and coils are designed to be of the same mass. The structure consists of a handle and two arms. The two arms are connected at one end and are free at the other, forming a U shape. One end of the handle is connected to the common ends of the two arms, and the other end is hinged with the adjusting screw after being clamped through a preload. The adjusting screw is connected with the thread of the supporting housing. The tuning fork, as a whole, is similar to an overhanging beam. The extension length of the tuning fork structure will change with the rotation of adjusting knob. To avoid the effects of magnetic fields, the whole structure is made of phosphor bronze. The stiffness of the two arms is greater than that of the handle. The magnet is a cylindrical Ndfeb magnet. The magnetization direction is along the axis of the cylinder. The coil is circular, with an inner diameter that is slightly larger than the outer diameter of the magnet. The central axes of the coil and magnet coincide and are fixed at the free ends of the two arms. Underneath the entire structure is the base, which also acts as the impact surface. Tuning fork structures encounter collision surfaces when stimulated by external stimuli. The geometric structure is shown in Figure 1.

**Figure 2:**
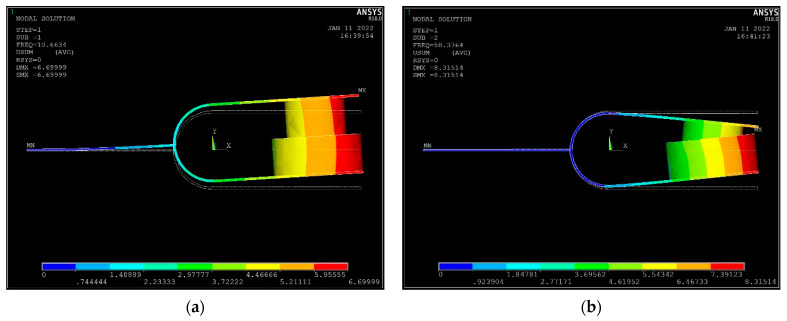
(**a**) The in-phase vibration mode and (**b**) the anti-phase vibration mode.

For the structure, only the first two vibration modes are considered. The first mode of vibration has a low frequency and easily resonates with low-frequency excitation, collecting more energy. Moreover, in the first mode, the tuning fork arm moves up and down with the handle, there is no relative displacement between the coil and the magnet, and no energy is converted into electricity. This is called the in-phase vibration mode, as shown in Figure 2a. In the second mode, the tuning fork handle does not move, and the two tuning fork arms are close to or far away from each other and have a high vibration frequency. This is called the anti-phase vibration mode, as shown in Figure 2b. The energy collected in the in-phase mode is transferred to the antiphase mode to generate electricity by the impact.

**Figure 3:**
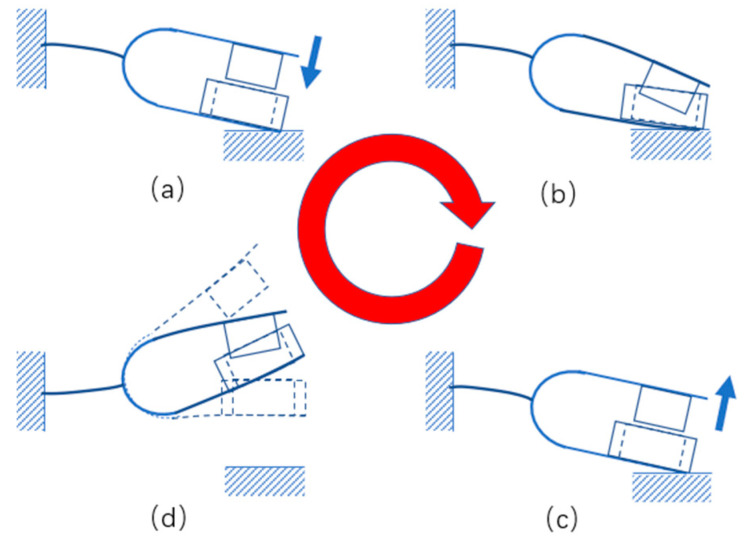
Schematic diagram of the movement process of the electromagnetic tuning fork energy harvester. (**a**) Impact, (**b**) maximum deformation, (**c**) moment of separation after impact and (**d**) vibration after separation.

In order to demonstrate the movement process of the energy harvester more clearly, its important movement positions are listed in Figure 3.

Compared with a piezoelectric tuning fork, the electromagnetic tuning fork not only retains the tuning fork structure through different vibration modes of frequency conversion function, but after replacing the sensitive mass of the piezoelectric tuning fork with the magnet and coil of the same mass, the energy harvester avoids the disadvantageous factor of the large internal resistance of the piezoelectric mode, reduces the internal resistance, and improves the output power. It has a higher energy density than the same-sized piezoelectric tuning fork energy harvester. The function of adjustable natural frequency is added. In the range of frequency regulation, energy capture can be realized, even if the excitation frequency changes in the environment, and it is always in the resonance state.

**Figure 4:**
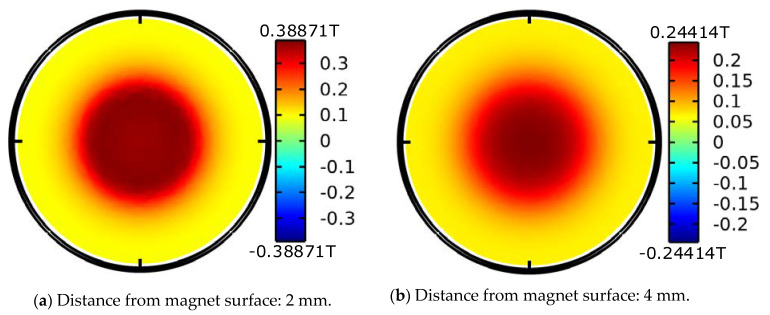

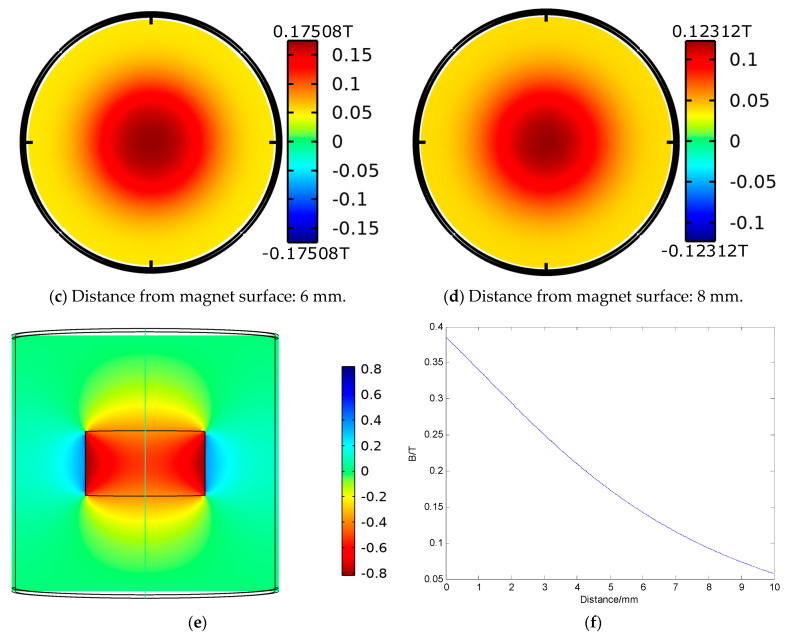
Magnetic cloud of a magnet (**a**–**e**) and axial magnetic field variation (**f**).

Figure 4a–d are the cloud images of magnetic field at 2, 4, 6 and 8 mm away from the magnet surface, respectively. As the distance increases, the maximum magnetic field intensity decreases from 0.38871 T in Figure 4a to 0.12312 T in Figure 4d. Figure 4e is the magnetic cloud image of the section where the magnet passes through the axis. Although the maximum magnetic field intensity is close to 0.85 T as shown in Figure 4e, the strongest point is inside the magnet. In order to understand the magnetic field in detail, the variation in magnetic field intensity along the axis of the magnet with distance is drawn in Figure 4f.

It can be seen from the figure that the magnetic field intensity decreases with increasing distance from the magnet, so the distance between the coil and the magnet is designed to be very small in the prototype design.

The basic working principle of the electromagnetic tuning fork energy harvester is that, under the action of excitation, it first swings up and down according to the first-order vibration mode and makes forced vibration. When the free end of the lower tuning fork arm moves to the impact surface, a collision occurs, the first-order vibration provides the initial speed of the collision, and the second-order vibration is excited, causing the magnet and coil to move relative to each other, and the magnetic flux through the coil changes and the kinetic energy is converted to electricity.

### 2.2. Electromechanical Coupling Dynamics Model

**Figure 5:**
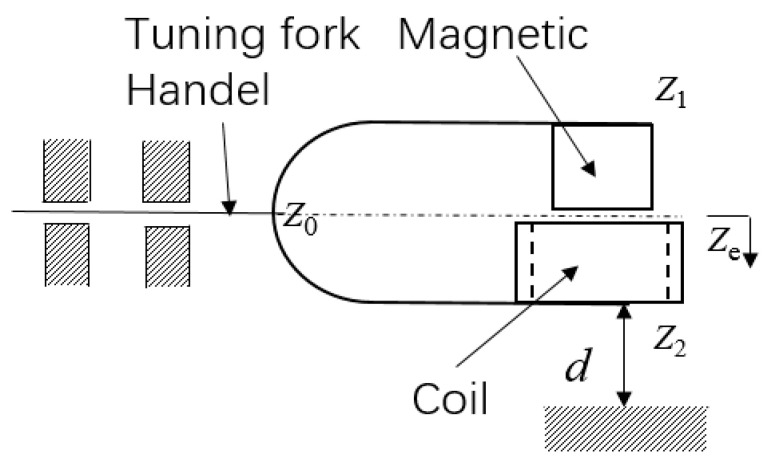
Schematic diagram of adjustable tuning fork structure.

Figure 5 shows the schematic electromagnetic tuning fork structure, where $z_{e}$ is the displacement of the tuning fork centreline and d is the impact gap. When the displacement was greater than the impact gap ($z_{e}>d$), the analysis was performed in two parts: the main movements and the high-frequency impact response.

The virtual work of the current-carrying coil in the magnetic field can be expressed as:

(1) δW=δ(IAB)

where I is the current in the coil, $A_{c}$ is the area of the coil, and B is the magnetic field intensity at the position of the coil.

For constant current and fixed area coils, there are:

(2) $$\delta W=IA_{c}\delta B$$

If current I is the induced current:

(3) $$I=\frac{A_{c}}{R_{c}+R_{m}}\frac{\partial B}{\partial t}$$

where $R_{c}$ and $R_{m}$ is the coil resistance and load resistance, respectively, and:

(4) $$\delta W=A_{c}\delta (IB)=\frac{{A_{c}}^{2}}{R_{c}+R_{m}}B\delta (\frac{\partial B}{\partial t})=\frac{{A_{c}}^{2}}{2(R_{c}+R_{m})}\delta (\frac{\partial B^{2}}{\partial t})$$

Therefore, the force in the relative motion direction $z_{I}$ between the magnet and the coil is:

(5) $$F_{I}=\frac{\partial W}{\partial z_{I}}=\frac{{A_{c}}^{2}}{2(R_{c}+R_{m})}\frac{\partial }{\partial z_{I}}(\frac{\partial B^{2}}{\partial t})$$

and because:

(6) $$\frac{\partial B^{2}}{\partial t}=\frac{\partial B^{2}}{\partial z_{I}}\frac{\partial z_{I}}{\partial t}$$

So:

(7) $$F_{I}=\frac{{A_{c}}^{2}}{2(R_{c}+R_{m})}\frac{\partial }{\partial z_{I}}(\frac{\partial B^{2}}{\partial z_{I}}\frac{\partial z_{I}}{\partial t})=\frac{{A_{c}}^{2}}{2(R_{c}+R_{m})}\frac{\partial^{2}B^{2}}{\partial z_{I}^{2}}{\dot{z}}_{I}$$

The tuning fork structure can be expressed by the following governing equations [30]:

(8) $$\{\begin{array}{l} 2m{\ddot{z}}_{e}+\frac{2cc_{0}}{c_{0}+2c}{\dot{z}}_{e}+\frac{2kk_{a}}{k_{a}+2k}z_{e}=2ma(t)\\ m{\ddot{z}}_{I}+(c+\frac{{A_{c}}^{2}}{2(R_{c}+R_{m})}\frac{\partial^{2}B^{2}}{\partial z_{I}^{2}}){\dot{z}}_{I}+kz_{I}=0 \end{array}$$

The first equation in Equation (8) is the in-phase vibration mode, and the second equation is the anti-phase vibration mode, where $z_{1}$ and $z_{2}$ are the displacement of magnet and coil, respectively, $z_{e}=\frac{1}{2}(z_{1}+z_{2})$ and $z_{I}=z_{1}-z_{2}$, m is the mass of the end of the magnet or coil, c is the damping coefficient of the tuning fork arm structure, k is the stiffness of the tuning fork arm, $k_{a}$ is the adjustable tuning fork handle structural stiffness, ω is the frequency of excitation, and a(t) is the environmental acceleration.

The second equation in Equation (8) shows that the damping of the system decreases with increasing external load resistance. 

At the beginning, the distance between the free end of the tuning fork and the collision surface is d, so as to make forced vibration.

The initial conditions are:

(9) $$z_{e}(0)=z_{e0},\;{\dot{z}}_{e}(0)={\dot{z}}_{e0}$$

The displacement and velocity of in-phase vibration are obtained from the first equation the first equation in Equation (8):

(10) $$\begin{array}{ll} z_{e}(t) & =e^{-\varsigma \omega_{n}t}[z_{e0}\cos\omega_{d}t+\frac{{\dot{z}}_{e0}+\varsigma \omega_{n}z_{e0}}{\omega_{d}}\sin(\omega_{d}t)]\\ & +B_{I}e^{-\varsigma \omega_{n}t}[\sin\varphi \cos(\omega_{d}t)+\frac{\omega_{n}}{\omega_{d}}(\varsigma \sin\varphi -\lambda \cos\varphi )\sin(\omega_{d}t)]+B_{I}\sin(\omega t-\varphi ) \end{array}$$

(11) $$\begin{array}{ll} {\dot{z}}_{e}= & -\varsigma \omega_{n}e^{-\varsigma \omega_{n}t}[z_{e0}\cos(\omega_{d}t)+\frac{{\dot{z}}_{e0}+\varsigma \omega_{n}z_{e0}}{\omega_{d}}\sin(\omega_{d}t)]\\ & +e^{-\varsigma \omega_{n}t}[-z_{e0}\omega_{d}\sin(\omega_{d}t)+({\dot{z}}_{e0}+\varsigma \omega_{n}z_{e0})\cos(\omega_{d}t)]\\ & -B_{I}\varsigma \omega_{n}e^{-\varsigma \omega_{n}t}[\sin\varphi \cos(\omega_{d}t)+\frac{\omega_{n}}{\omega_{d}}(\varsigma \sin\varphi -\lambda \cos\varphi )\sin(\omega_{d}t)]\\ & +B_{I}e^{-\varsigma \omega_{n}t}[-\omega_{d}\sin\varphi \sin(\omega_{d}t)+\omega_{n}(\varsigma \sin\varphi -\lambda \cos\varphi )\cos(\omega_{d}t)]\\ & +B_{I}\omega \cos(\omega t-\varphi ) \end{array}$$

where:

$\omega_{n}=\sqrt{\frac{2kk_{a}}{m(k_{a}+2k)}}$, $\omega_{d}=\omega_{n}\sqrt{1-\varsigma^{2}}$, $\varsigma =\frac{cc_{0}}{\omega_{n}m(c_{0}+2c)}$, $F_{0}=2ma$, $\lambda =\frac{\omega }{\omega_{n}}$,

$B_{I}=\frac{2F_{0}kk_{a}}{(k_{a}+2k)\sqrt{{\left(1-\lambda^{2}\right)}^{2}+{\left(2\varsigma \lambda \right)}^{2}}}$, $\varphi =\tan^{-1}(\frac{2\varsigma \lambda }{1-\lambda^{2}})$

When the in-phase vibration displacement is first reached, d, the time is $t_{d}$. According to Equation (11), the velocity at this time can be obtained by using MATLAB iteration. Therefore, the initial conditions of the anti-phase mode can be obtained:

(12) $$z_{I}(0)=z_{I0},\;{\dot{z}}_{I}(0)={\dot{z}}_{e0}$$

The displacement and velocity of anti-phase vibration are obtained from the second equation in Equation (8).

(13) $$z_{I}=Ae^{-\varsigma \omega_{n}t}\sin(\omega_{d}t+\theta )$$

(14) $${\dot{z}}_{I}=Ae^{-\varsigma \omega_{n}t}[-\varsigma \omega_{n}\sin(\omega_{d}t+\theta )+\omega_{d}\cos(\omega_{d}t+\theta )]$$

where:

$\omega_{na}=\sqrt{\frac{k}{m}}$, $\omega_{da}=\omega_{na}\sqrt{1-\varsigma_{a}^{2}}$, $\varsigma_{a}=\frac{c+\frac{A^{2}}{2(R_{c}+R_{m})}\frac{\partial^{2}B^{2}}{\partial z_{I}^{2}}}{2m\omega_{na}}$

$A=\sqrt{z_{I0}^{2}+{\left(\frac{{\dot{z}}_{e0}+\varsigma \omega_{na}z_{I0}}{\omega_{da}}\right)}^{2}}$, $\theta =\tan^{-1}(\frac{\omega_{d}z_{I0}}{{\dot{z}}_{e0}+\varsigma \omega_{n}z_{I0}})$

If $k_{a}$ is much smaller than k, the tuning fork structure first performs low-frequency forced vibration according to in-phase vibration mode to capture and accumulate environmental energy, and then performs high-frequency free attenuation vibration through shock excitation of the antiphase vibration mode of the tuning fork, and converts the captured energy into power generation by using the relative motion of the magnet and coil.

In addition to the main motion, due to the impact between the tuning fork structure and the impact surface, a high-frequency impact response is generated in the impact process. 

The high-frequency impact response is caused by the local elastic deformation of the material at the impact site. The dynamic equation of the tuning fork arm tip mass can be written as [30]:

(15) $$\{\begin{array}{l} m{\ddot{z}}_{1}+\frac{1}{2}kz_{1}+(F_{I1}+F_{I2})=0\\ m\ddot{\delta }+\frac{1}{2}\varepsilon k_{m}(\delta -z_{1})=-k_{m}\delta^{n} \end{array}$$

where $k_{m}$ is the deformation stiffness of the tip coil, δ is the displacement of mass in the impact process, n is a constant related to the shape of the mass structure, and $F_{I1}$ and $F_{I2}$ are the electromagnetic forces of magnets and coils. $\varepsilon =\frac{k}{k_{m}}$ is a small quantity.

The initial conditions are:

(16) $${\dot{z}}_{1}(t=0)=\dot{\delta }(t=0)=v_{0}$$

The multiscale perturbation method is used to analyse and solve the problem. The first-order approximate solution is:

(17) $$\delta =\frac{v_{0}}{\omega_{m}}\cos\frac{1}{2}\frac{\omega^{2}}{\omega_{m}}t\sin\omega_{m}t-\frac{1}{2}\frac{\varepsilon \omega_{m}{}^{2}}{\omega_{m}{}^{2}-\omega^{2}}(\frac{v_{0}}{\omega_{m}}\cos\omega_{m}t-\frac{v_{0}}{\omega }\sin\omega t)$$

(18) $$\dot{\delta }=\frac{v_{0}}{\omega_{m}}\cos\frac{1}{2}\frac{\omega^{2}}{\omega_{m}}[\sin(\omega_{m}t)+\omega_{m}t\cos\omega_{m}t]+\frac{1}{2}\frac{v_{0}\varepsilon \omega_{m}{}^{2}}{\omega_{m}{}^{2}-\omega^{2}}[\sin\omega_{m}t+\cos\omega t)]$$

It is observed that if n=1, its impact period is $\tau =\frac{2\pi }{\omega_{m}}$, where $\omega_{m}=\sqrt{\frac{k}{m}}$ is the frequency of high-frequency impacts.

Magnets and coils are mounted on the free ends of both tuning fork arms, anti-phase vibration and high-frequency vibrations from the impact determine the relative displacement of the magnet and coil, and in-phase vibration provides the initial velocity for the relative displacement of the magnet and coil. 

The total relative displacement between magnet and coil is:

(19) $$\begin{array}{ll} z(t)=z_{I}+\delta & =Ae^{-\varsigma \omega_{n}t}\sin(\omega_{d}t+\theta )\\ & +\frac{v_{0}}{\omega_{m}}\cos\frac{1}{2}\frac{\omega^{2}}{\omega_{m}}t\sin\omega_{m}t-\frac{1}{2}\frac{\varepsilon \omega_{m}{}^{2}}{\omega_{m}{}^{2}-\omega^{2}}(\frac{v_{0}}{\omega_{m}}\cos\omega_{m}t-\frac{v_{0}}{\omega }\sin\omega t) \end{array}$$

(20) $$\begin{array}{ll} \dot{z}(t)={\dot{z}}_{I}+\dot{\delta } & =Ae^{-\varsigma \omega_{n}t}[-\varsigma \omega_{n}\sin(\omega_{d}t+\theta )+\omega_{d}\cos(\omega_{d}t+\theta )]\\ & +\frac{v_{0}}{\omega_{m}}\cos\frac{1}{2}\frac{\omega^{2}}{\omega_{m}}[\sin(\omega_{m}t)+\omega_{m}t\cos\omega_{m}t]+\frac{1}{2}\frac{v_{0}\varepsilon \omega_{m}{}^{2}}{\omega_{m}{}^{2}-\omega^{2}}[\sin\omega_{m}t+\cos\omega t)] \end{array}$$

The change in magnetic field along the axis of the cylinder can be expressed as [10]:

(21) $$\frac{dB_{M}}{dz_{I}}=\frac{B_{r}R_{B}{}^{2}}{2}\{\frac{1}{{\left[{\left(z_{I}+L_{B}\right)}^{2}+R_{B}{}^{2}\right]}^{3/2}}-\frac{1}{{\left(z_{I}{}^{2}+R_{B}{}^{2}\right)}^{3/2}}\}$$

The relation between the output voltage and time can be expressed using Equation (22) [10]:

(22) $$v_{I}(t)=-\frac{dB_{M}}{dz_{I}}\dot{z}(t)A_{c}$$

where $v_{I}(t)$ is the induced voltage, $A_{c}$ is the area of the coil, $B_{M}$ is the magnetic flux density, $B_{r}$ is the flux density, $R_{B}$ is the radius of the magnet, and $L_{B}$ is the height of the magnet.

## 3. Simulation and Experiment

In order to verify the results of theoretical analysis, the relation between voltage and time is compared by calculation and experiment.

**Figure 6:**
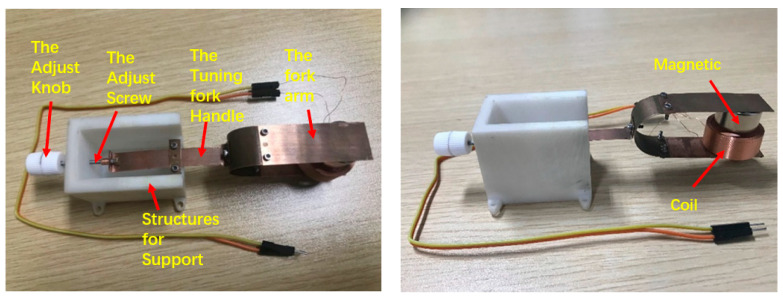
Prototype adjustable frequency tuning fork electromagnetic energy harvester.

According to Figure 1, the adjustable frequency tuning fork electromagnetic energy harvester was designed and manufactured. Figure 6 shows a photograph of the prototype. The parameters of the electromagnetic energy harvester are shown in Table 1.

**Figure 7:**
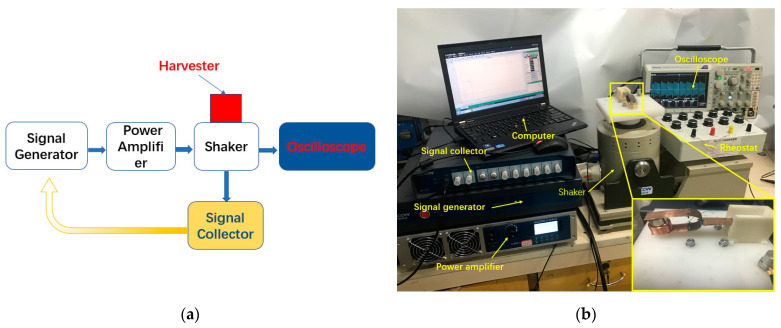
(**a**) Schematic diagram of experimental device; (**b**) experimental prototype and test system.

Figure 7 shows the setup for testing the tuning fork energy harvester. This experimental testing equipment forms a closed loop system, which can test the experimental prototype truly and accurately. The control signal of the experimental device is generated by the signal generator, amplified by the power amplifier, and then transmitted to the vibration exciter to control the vibration of the exciter. The signal collector collects the output signal and returns it to the signal generator to calibrate the output signal. The harmonic excitation is used as the input signal of the system. The accelerometer is fixed on the excitation platform to monitor the input vibration of the energy harvester. The oscilloscope is connected to the energy harvester to measure and record the output characteristics of the energy harvester.

According to the calculation of the parameters in Table 1, a prototype was created and experimental tests were carried out.

**Figure 8:**
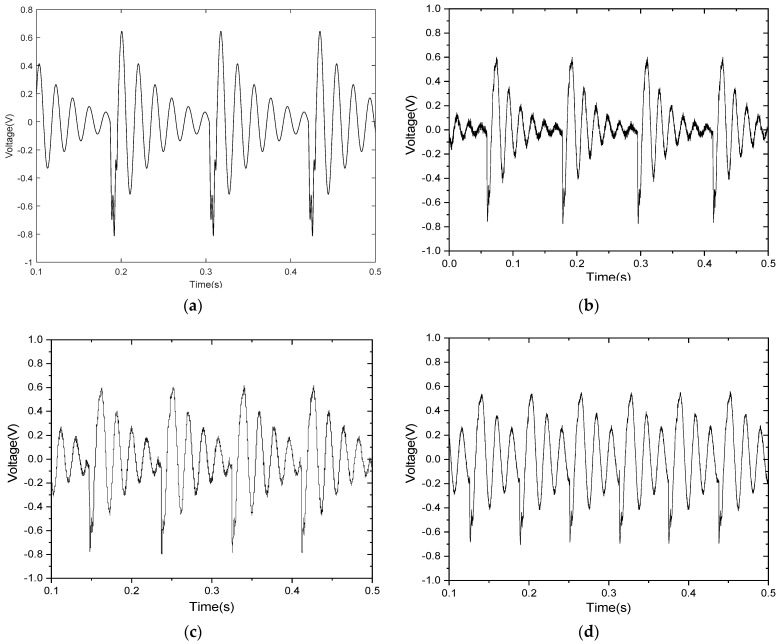
(**a**) The calculated voltage waveform and (**b**) the measured instantaneous voltage waveform; ((**a**,**b**) are obtained under the condition that L_0_ = 3.5 cm, impact clearance is 1.0 cm, and acceleration is 0.5 g) (**c**,**d**) are experimental results (L_0_ is 3.0 cm and 2.5 cm, respectively, the spacing is 1.0 cm, and the external load resistance is 25 Ω).

When the excitation acceleration is 0.5 g and the initial impact distance between the free end of the lower arm of the tuning fork and the impact surface is 1.0 cm, the waveform of voltage changing with time can be obtained from Equation (22), as shown in Figure 8a. The corresponding experimental curve is shown in Figure 8b.

As shown in Figure 8b, from the oscilloscope-recorded data, the time between the two collisions is 0.1087 s, and every two free attenuation amplitude intervals is 0.0167 s. Thus, the energy-harvesting frequency is 9.2 Hz and the power-generation frequency is 60 Hz. The impact period has obvious high-frequency vibration characteristics. During the separate period, the tuning fork is in free vibration. The separate period vibration output voltage decays exponentially with time. The vibration attenuates freely six times and then enters the next impact period.

The comparison between the theoretical and experimental results shows that the experimental results basically coincide with the theoretical design. 

**Figure 9:**
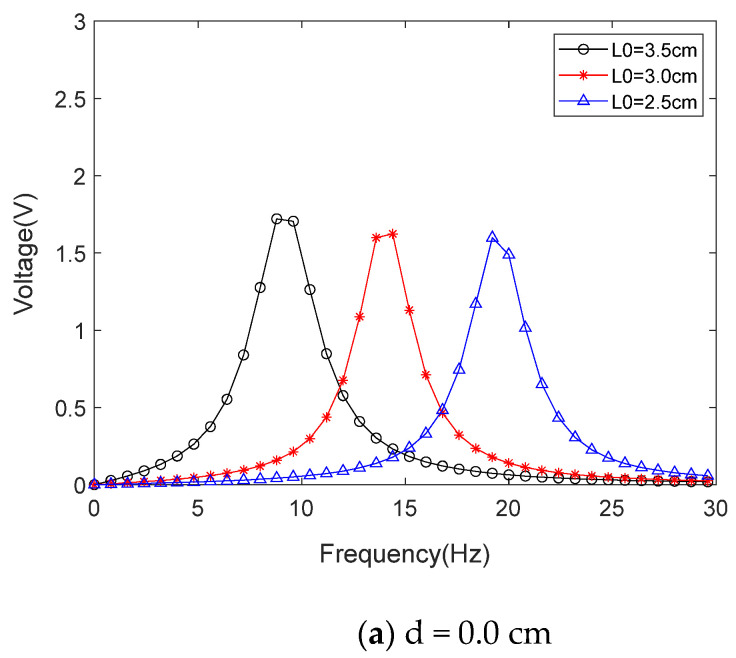

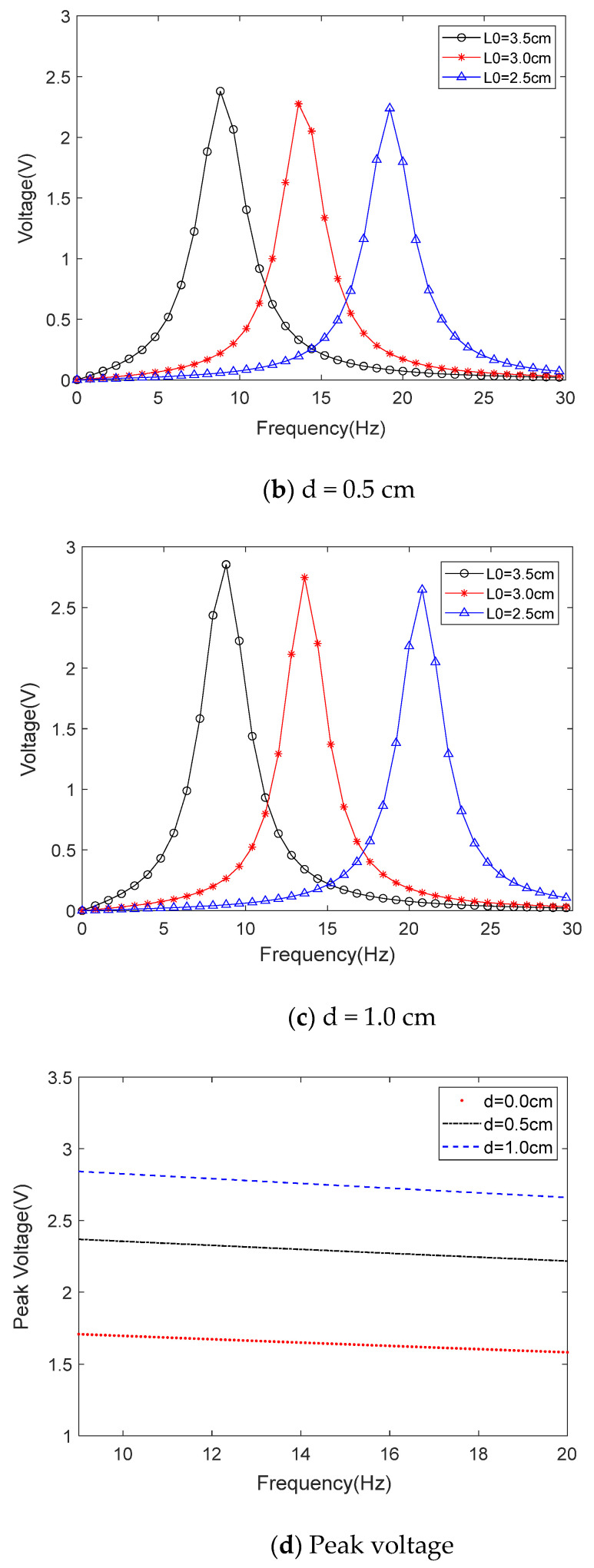
The simulated voltage under various impact gaps (L_0_ is the length of the tuning fork handle). (**a**–**c**) are three sweep curves under certain L_0_ conditions; (**d**) is the change in frequency and peak voltage generated by adjusting L_0_.

Furthermore, according to Equation (10), for each determined ω, there is a ${\dot{z}}_{e0}$, and the amplitude of the anti-phase vibration velocity can be obtained from Equation (14). The amplitude of output voltage can be obtained from Equation (22). Therefore, MATLAB can be used to obtain the amplitude–frequency response curve of the energy harvester, as shown in Figure 9.

**Figure 10:**
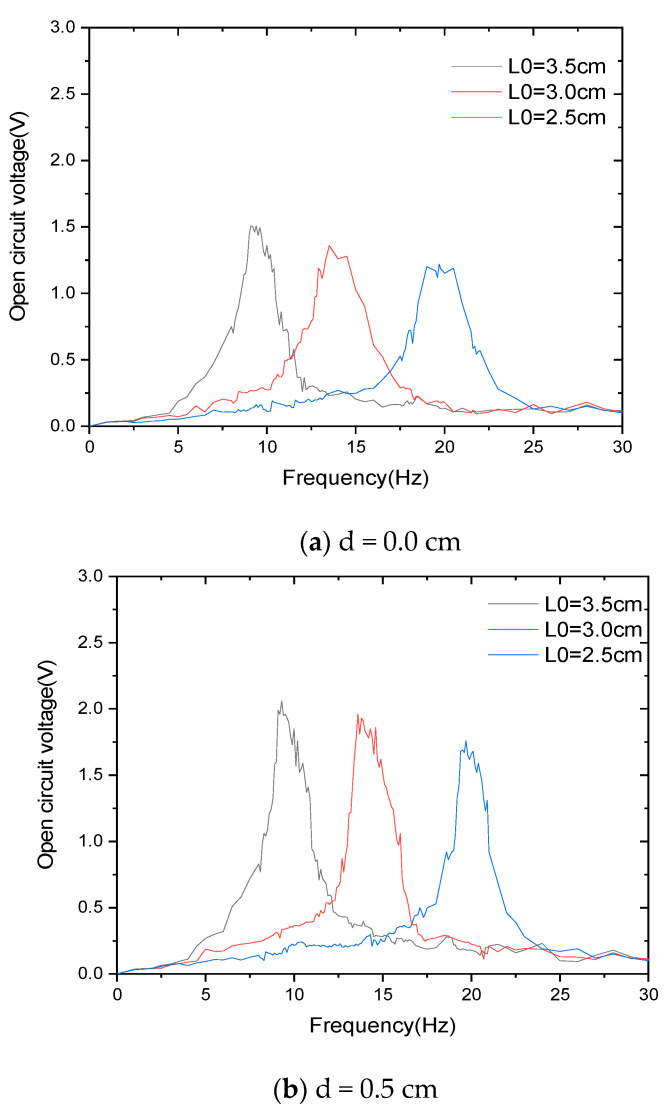

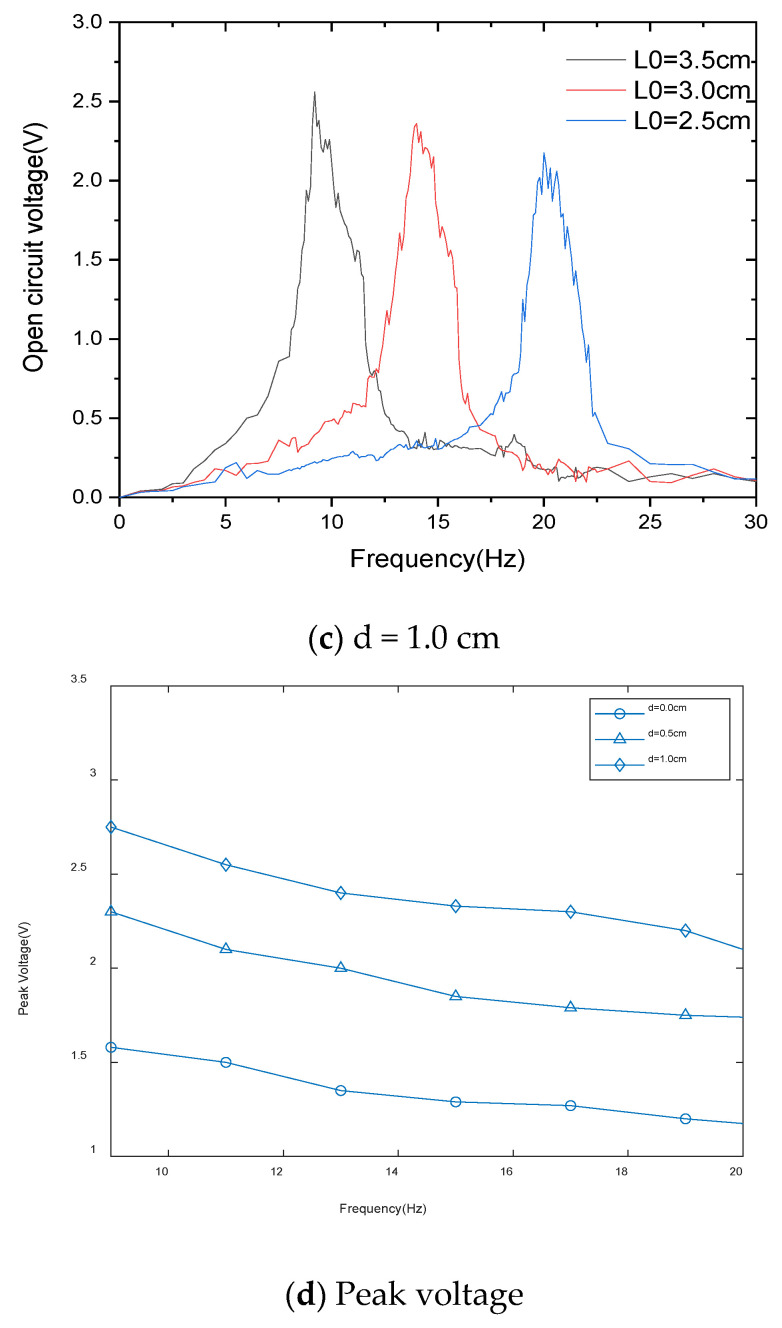
The measured voltage under various impact gaps.

To test the theoretical curve, experiments were carried out under the same conditions, and the results are shown in Figure 10.

Figure 10 shows the experimental tests of adjusting the tuning fork handle length in a range of 2.5–3.5 cm at different impact spacings. Through frequency sweep experimental tests, it was found that, when adjusting the frequency, the peak output frequency range of the energy harvester was 9.2–21.1 Hz. In this frequency range, the energy harvester can output power in a resonant state. Open circuit voltages are all greater than 1.2 V. Figure 10a shows that the scanning voltage under the impact clearance is 0 cm, and the energy harvester is within the adjustment range (2.5–3.5 cm). With the decrease in the length of the tuning fork handle, the resonant frequency increases and the peak voltage decreases from 1.51 to 1.2 V, which is a slight but not significant decrease. Figure 10b shows that the scanning voltage under the impact clearance is 0.5 cm and the energy harvester is within the adjustment range. The resonant state voltage of the energy harvester has been reduced from 2.16 to 1.75 V. Figure 10c shows that the scanning voltage under the impact clearance is 1.0 cm, and the energy harvester is within the adjustment range (2.5–3.5 cm). The power harvester’s resonant state voltage reduced from 2.57 to 2.2 V, which is again a small drop. Figure 10d shows the change in frequency and peak voltage generated by adjusting L_0_.

We designed the adjustment range of the tuning fork handle length as 2.5–3.5 cm, and peak voltage varies across different resonant states of the energy harvester by very little.

The peak voltage of the energy harvester under different excitation frequencies was tested, and the results are shown in Figure 10d. The voltage increased with the increase in the impact gap.

**Figure 11:**
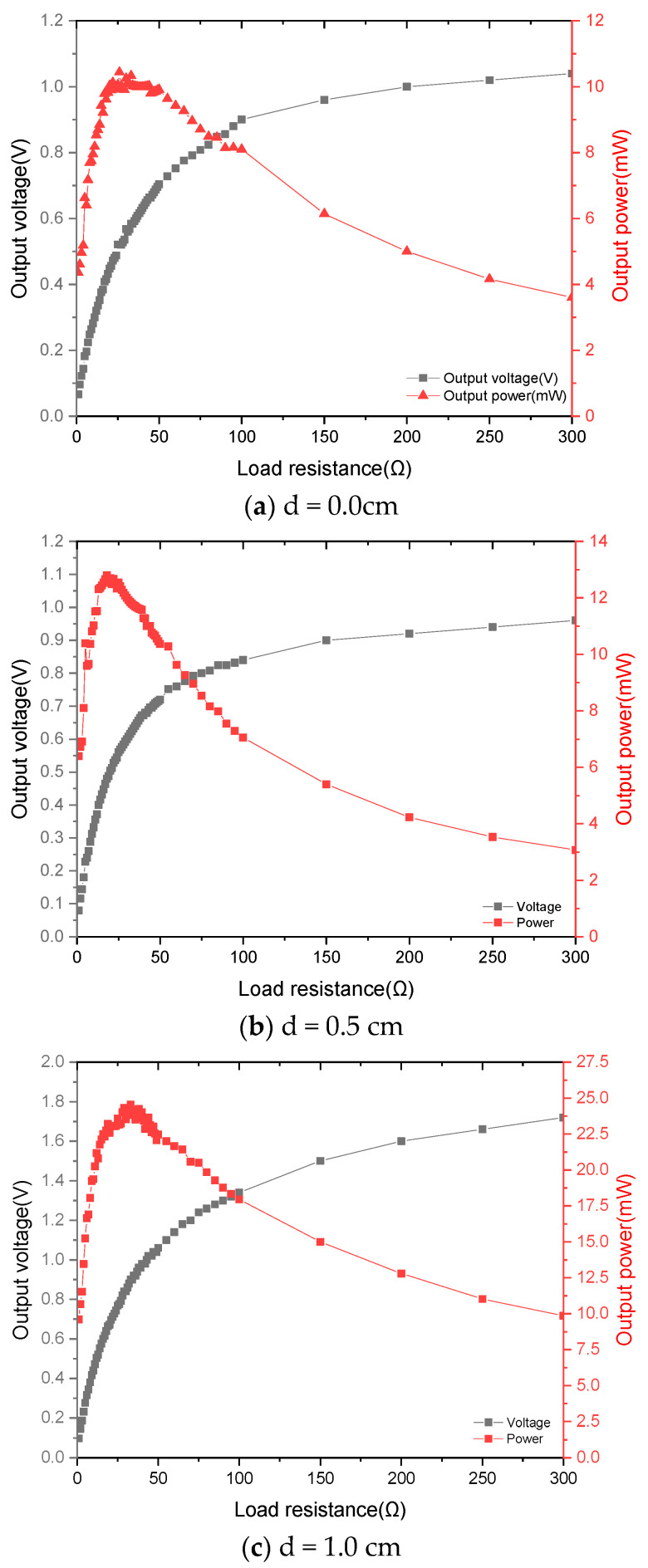
The voltage and power delivered to the different loads ((**a**) d = 0.0 cm; (**b**) d = 0.5 cm; (**c**) d = 1.0 cm) when the length of the tuning fork handle is 3.5 cm.

Figure 11 shows the voltage and power delivered to the load. By changing the load resistance at a frequency of 9.2 Hz at different impact clearances, the optimal load resistance for maximum power transmission was determined to be 25 Ω. It can be found from the curve of voltage changing with load resistance that the voltage across the load increases as the load resistance increases. When the load resistance matches the source resistance, the output power is maximum, that is, when the external load resistance is equal to the source resistance, the output power is maximum. In the experiments, when the excitation acceleration is 0.5 g, the impact clearance is 1.0 cm, and the excitation frequency is 9.2 Hz, a maximum power of 23.59 mW is delivered to the 25 Ω resistor.

In order to compare with other EMES, the output power of other EMES and EMES proposed in this paper is listed in Table 2. As can be seen from Table 2, the output power of EMES proposed in this paper has higher output power than other EMES.

## 4. Power Supply Experiment for Electrical Appliances

**Figure 12:**
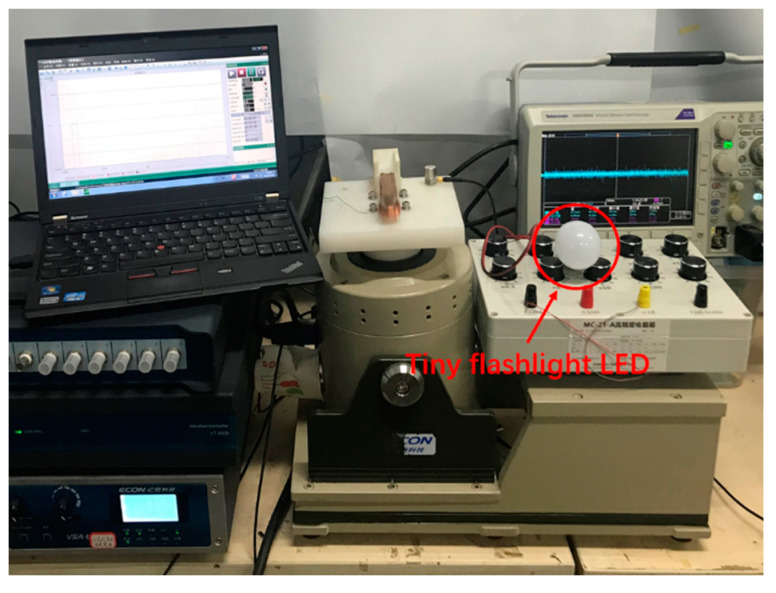
Circuit diagram used to power tiny flashlight LED (a=0.5 g, d=1.0 cm).

To demonstrate a practical application, a small flashlight LED with a diameter of 46.5 mm was connected to the circuit and powered by an energy harvester. The tuning fork energy harvester is connected to the LED lamp for the power experimental test, as shown in Figure 12.

**Figure 13:**
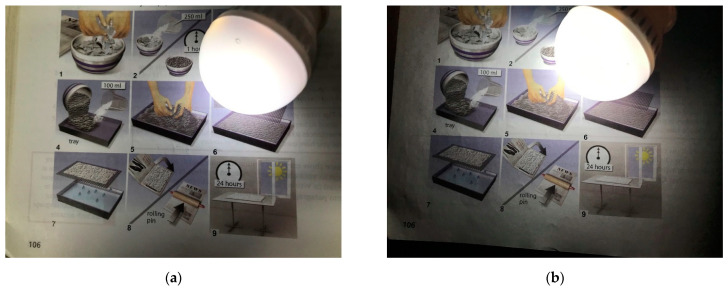
A tiny flashlight LED powered by the tuning fork energy harvester. (**a**) When indoor lighting is on; (**b**) when the indoor light is turned off.

After connecting as shown in Figure 12, under an excitation acceleration of 0.5 g, the impact gap was 1.0 cm, and the excitation frequency was 9.2 Hz. The experimental results are shown in Figure 13. The tuning fork energy harvester can output stably and make a tiny flashlight LED work.

## 5. Conclusions

In this paper, a frequency-adjustable tuning fork electromagnetic energy harvester is presented. The natural frequency can be easily and quickly adjusted without any changes to the structure. Therefore, it has better applicability with varying excitation frequency. By adjusting the stiffness of the tuning fork handle, the first-order vibration mode frequency of the structure can be changed. When close to or resonant with the changing excitation frequency in the environment, more energy can be obtained. By means of impact, energy is transferred to higher-order vibration modes and converted into electrical energy.

Theoretical analysis and experimental verification of the energy harvester under different excitation frequencies were carried out. The results show that the frequency regulation range of the energy collector is 9.2–20.1 Hz. Frequency modulation has little effect on the output voltage of the energy harvester and can realize the resonance of any value within the adjustable frequency range. When the acceleration is 0.5 g and the impact spacing is 1.0 cm, the resonance frequency is 9.2 Hz, the peak voltage is 2.6 V, and the peak power is 23.59 mW. Compared with the same-sized piezoelectric tuning fork energy harvester, not only is the frequency adjustable, but the peak output power increases by 14.85 mW the power density increases by 169.88%.

In addition, the power supply of the electromagnetic tuning fork energy collector is tested. The results show that the electromagnetic tuning fork energy collector can provide stable electric energy to a micro-flashlight, such that it can work normally and stably. The electromagnetic tuning fork energy collector can be used as a stable power source for small electrical appliances. The device has the potential to be used in low-frequency and frequency-changing vibration environments, providing power for wearable devices or as a charger.

## Figures and Tables

**Table 1:** Material properties and structural parameters of the adjustable frequency tuning fork electromagnetic energy harvester.

Parameter	Value
Diameter of magnet (NdFeB)	17 mm
Thickness of magnet (NdFeB)	10 mm
Remanent (Br)	1.2 T
Coil (copper) number of turns	500
Outer diameter of coil (copper)	24 mm
Inner diameter of coil	20 mm
Young’s modulus of beryllium bronze (Ya)	133 GPa
Size of the handle (l_0_ × b_0_ × h_0_)	35~25 mm × 8 mm × 0.6 mm
Size of the arm (l_a_ × b_a_ × h_a_)	30 mm × 16 mm × 0.5 mm
The radius of the arc (R)	10 mm
Stiffness coefficient of the arm (k)	1420 N/m
Stiffness coefficient of the handle (k_a_)	65 N/m
Damping ratio (ς)	0.015

**Table 2:** Comparison with other state-of-the-art EMEHs.

References	Peak Power
Elvin et al. [1]	1.7 μW
Li et al. [30]	12 mW
Iqbal et al. [10]	175 μW
Dallago. [31]	6 mW
Proposed	23.59 mW

## Data Availability

The data that support the findings of this study are available from the corresponding author upon reasonable request.
